# Association between dietary calcium and depression among American adults: National health and nutrition examination survey

**DOI:** 10.3389/fnut.2023.1042522

**Published:** 2023-02-09

**Authors:** Xia Shen, Xue Gu, Yuan-Yuan Liu, Long Yang, Meng Zheng, Lei Jiang

**Affiliations:** ^1^Wuxi Medical College, Jiangnan University, Wuxi, China; ^2^Department of Pediatric Cardiothoracic Surgery, The First Affiliated Hospital of Xinjiang Medical University, Ürümqi, China; ^3^The Fifth Medical Center of People’s Liberation Army (PLA) General Hospital, Beijing, China; ^4^Department of Radiology, The Convalescent Hospital of East China, Wuxi, China

**Keywords:** depression, dietary calcium, calcium, adults, National Health and Nutrition Examination Survey (NHANES)

## Abstract

**Background:**

There is only limited evidence for an association between calcium (Ca) and depression, and the relationship was inconsistent. Therefore, the aim of this study was to assess the relationship between dietary Ca and the risk of depressive symptoms in individuals over the age of 18 in the US.

**Methods:**

We extracted 14,971 participants from the US National Health and Nutrition Examination Survey (NHANES) 2007–2016 to probe their associations. Dietary Ca intake was measured through 24 h dietary recall method. Patients with the Patient Health Questionnaire-9 (PHQ-9) ≥ 10 scores were believed to have depressive symptoms. The association between dietary Ca and depressive symptoms was investigated using multivariate logistic regression, sensitivity analysis, and restricted cubic spline regression.

**Results:**

In this study, 7.6% (1,144/14,971) of them had depressive symptoms. After adjusting for sex, age, race, poverty to income ratio (PIR), marital status, education, body mass index (BMI), caffeine intake, carbohydrates intake, total energy intake, smoking status, alcohol consumption, physical activity, diabetes, hypertension, severe cardiovascular disease (CVD), cancer, serum vitamin D, serum Ca, and Ca supplement, the adjusted ORs value [95% confidence interval (CI)] of depression for the lowest category (Q1 ≤ 534 mg/day) vs. Q2–Q4 of Ca intake were 0.83 (0.69–0.99), 0.97 (0.65–0.95), and 0.80 (0.63–0.98) with the *p* for trend (*p* = 0.014). The relationship between dietary Ca intake and depressive symptoms was linear (non-linear *p* = 0.148). None of the interactions were significant except among races (*p* for interaction = 0.001).

**Conclusion:**

Association between dietary Ca and the prevalence of depressive symptoms in US adults. And Ca intake was negatively associated with the risk of depressive symptoms. As Ca intake increased, the prevalence of depressive symptoms decreased.

## 1. Introduction

Depression remains one of the most prevalent and disabling biopsychosocial conditions in people (1–3). Nearly 300 million people of all ages worldwide suffer from this disease (4). In 2017, nearly 17.3 million adults in the United States were reported to have experienced at least one major depressive episode, with a prevalence of about 7.1% (5). The occurrence of depression can seriously reduce the quality of life and also bring many burdens, especially for the elderly (6). Based on this fact, it is necessary to identify strategies and methods that are effective in preventing depression.

Lifestyle factors are considered to be one of the important mediators of the pathophysiology associated with mental disorders (7). An increasing number of epidemiological studies have shown an association between diet and mental health (8). Several dietary factors such as coffee consumption, carbohydrates, and energy intake are thought to be associated with the risk of depression (9–11). Micronutrients are essential dietary elements that the body needs in varying amounts, including vitamins and trace minerals, to regulate physiological processes to maintain body function and health (12). Dietary minerals play an important role in the occurrence of cell regeneration processes, immune support, and antioxidants in the human body (13, 14). Appropriate requirements for essential dietary micronutrients can minimize the risk of overnutrition or deficiency (15). Moreover, dietary minerals may contribute to the development of disease pathology and course when their balance is impaired in the body (16, 17). Recently, more attention has been paid to the link between trace minerals such as zinc, copper, magnesium, and iron and mental disorders (8, 18–21).

Calcium (Ca) is one of the most essential elements, it’s not made by the body, and it has to be supplied exogenously, mainly through food (22) getting it can from foods such as beans, milk and dairy products, fish and seafood, vegetables, and algae. At the same time, vitamin D supplementation also helps the body absorb Ca better. In addition to getting Ca from food and supplements, exposure to sunlight is also a good way to get it. The ultraviolet light in the sun facilitates the synthesis of vitamin D, which promotes the absorption of Ca in the small intestine, thus achieving the effect of indirect Ca supplementation. Ca is one of the most essential elements and is also an activator of many enzymes in the body, promoting the normal functioning of the body’s organs (23). One study indicated that Ca leakage from ryanodine receptors (RyRs) was associated with neuronal firing and cognitive performance in aged macaques (24). Ca intakes are reported to be below the recommended levels in many countries in the world (25–27). Many countries in Asia have average dietary Ca intakes below 500 mg/day, most countries in Africa and South America have Ca intakes between 400 and 700 mg/day, and only the Nordic countries have national Ca intakes above 1,000 mg/day (28). Ca deficiency is thought to be associated with the development of many diseases, including osteoporosis, high blood pressure, diabetes, and heart disease (29–32).

Clinical studies have shown that Ca is associated with a mental disorders (33, 34). Rare studies have directly examined the relationship between Ca intake and depression separately (35, 36). Therefore, the purpose of this study was to examine the relationship between dietary Ca intake and depressive symptoms in a population of non-hospitalized American adults. We hypothesized that higher dietary Ca intake would be inversely related to depressive symptoms.

## 2. Materials and methods

### 2.1. Data sources and study population

**FIGURE 1:**
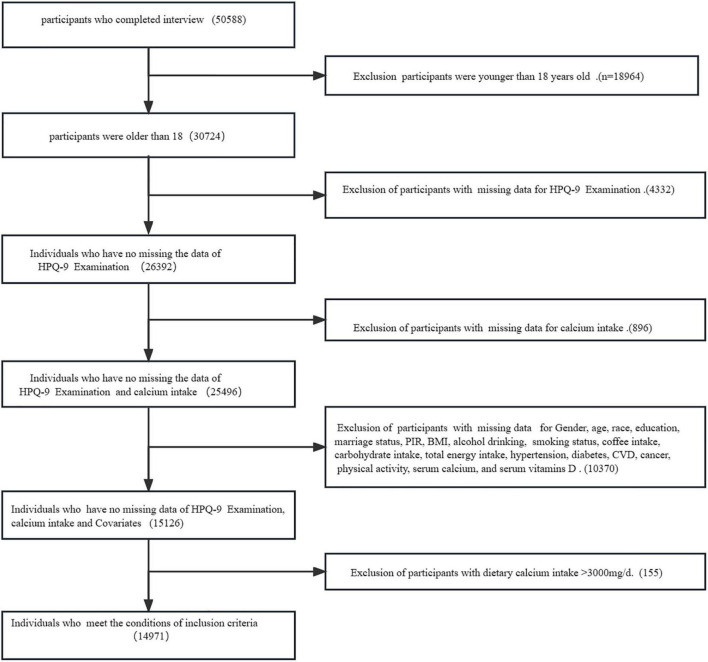
The flow chart of inclusion and exclusion of the participants in the study.

We used data from the National Health and Nutrition Examination Survey (NHANES) 2007–2016 database, and people older than 18 years who participated and completed the survey mobile examination center (MEC) were enrolled in our study (37). It uses a multi-stage stratified approach to assess the health and nutritional status of non-institutionalized Americans (38). Individuals lacking data on depression screening and Ca intake were excluded. It also excluded missing data: demographic and lifestyle behavior variables [sex, age, race, poverty to income ratio (PIR), marital status, education, body mass index (BMI), caffeine intake, carbohydrates, total energy intake, smoking, alcohol consumption, physical activity], chronic diseases [diabetes, hypertension, severe cardiovascular disease (CVD), cancer], and laboratory data (serum vitamin D, serum Ca), as shown in Figure 1. We also excluded those with extremely high Ca values to assess the impact of these potentially mismeasured outliers on our results (99th percentile, dietary Ca intake > 3,000 mg/day, *n* = 155).

The study was approved by the National Center for Health Statistics (NCHS) and the Research Ethics Review Board (39), and the ethics approval number is Protocol #2005-06 and Protocol #2011-17. Our study is based on publicly available data from NHANES, and the data are analyzed and extracted from 12–30 July, 2022. All details can be found on the official website: https://wwwn.cdc.gov/nchs/nhanes/search/default.aspx.

### 2.2. Depressive symptoms assessment

Depression symptoms is screened through the Patient Health Questionnaire-9 (PHQ-9) program (40), and the individual interviews were conducted at the mobile screening center using computer assistance. Approximately 5% of the interview information was recorded and reviewed for appropriate quality control. The PHQ consisted of nine depressive mood-related questions according to the Diagnostic and Statistical Manual, Fourth Edition, diagnostic criteria for major depressive disorder (41). It consists of nine questions (42), each of which has a maximum score of 3, with a total maximum score of 27. A total score greater than 10 is considered to be suffering from depressive symptoms, or it is a non-depressed category.

### 2.3. Dietary assessment

Dietary consumption data were collected from the 24 h before the interview (midnight to midnight). The type and amount of food and beverages consumed (43), as well as the number of nutrients and other food components, were validly assessed, including total energy, carbohydrates, coffee, and Ca. Data from the dietary interview component were collected by the NCHS at the U.S. Department of Agriculture (DHHS), which is responsible for survey sample design and data collection. The USDA’s Food Surveys Research Group (FSRG) was responsible for the dietary data collection methodology, maintenance of the database used to code and process the data, and data review and processing (44). Purpose of conducting dietary interviews was to obtain detailed dietary intake information from NHANES participants. And we chose the first day 24-recall diet interview data in our study.

### 2.4. Covariates assessment

Based on the literature (35, 45–50) and clinical practical implications we used the following covariates, including age, sex, race and ethnicity, education level, PIR, BMI, marital status, diabetes, hypertension, severe CVD, cancer/malignancy, total energy intake, carbohydrate intake, caffeine intake, serum Ca, Ca supplements, smoking status, alcohol consumption status, and physical activity. We divided the participants into five racial and ethnic groups. Mexican American, non-Hispanic Black, non-Hispanic White, other Hispanic, and other (including multiracial). Educational level was categorized into three levels {below high school, high school, and high school or grad/[General education development diploma (GED)]/equivalent} (51). We classified household income into the following four levels based on the proportion of household income in poverty: poor (PIR < 1), near poor (1 ≤ PIR < 2), moderate income (2 ≤ PIR < 4), and high income (PIR ≥ 4) (52). BMI weight divided by height squared, followed by classification according to criteria: low weight (<18.5), normal weight (18.5 ≤ BMI < 25), overweight (25 ≤ BMI < 30), and obesity (≥30) (53, 54). Marital status was still classified according to the NHANES: married, widowed, divorced, single, never married, living with a partner. Diabetes mellitus was defined as having been diagnosed by a physician (55). Hypertension and cancer were also based on self-reported hypertension or cancer (5, 56). Severe CVD was based on having a doctor’s diagnosis of coronary heart disease or heart attack or stroke (57).

Dietary data were obtained according to the 24-h dietary questionnaire in NHANES. Smoking status was categorized into the following three groups: never smoked (smoked less than 100 cigarettes in life), former (smoked at least 100 cigarettes in life and smoke, not at all), and current smoker (smoked more than 100 cigarettes in life and smoke some days or every day) (56). Drinking status was defined based on whether or not it was greater than 12 drinks in a year, and those who answered “yes” were drinking, otherwise, they were not. Physical activity is measured based on [Metabolic equivalent task (MET)] scores (58). Weekly as well as monthly exercise is converted to daily activity. For example, the met scores for vigorous-intensity work and vigorous recreational activities are calculated by multiplying by 8. Moreover, you multiply cycling or walking, moderate-intensity activity, and moderate recreational activity by four to get its MET scores.

### 2.5. Statistical analyses

The study was designed strictly according to the STROBE guidelines (59). All analyses were performed using the statistical software R (The R Foundation) and Free Statistics (Free Statistics 1.6) (60, 61). Demographic and clinical data were described by mean ± standard deviations, and frequency (percentages). Depending on whether the distribution was normal (approximately normal) or skewed, the distribution was processed using the one-way ANOVA and Kruskal–Wallis test for the relative response data. We stratified the analyses of dietary Ca intake in the following categories according to four quartiles: 534≤, 535–817, 818–1,190, and ≥1,191 mg/day.

Single sample *T*-test was used to compare the corresponding age group average values of magnesium intake and population recommended dietary allowances (RDAs). Univariate and multivariate logistic regression were used to investigate the relationship between Ca intake and the risk of depressive symptoms. Model 1 was unadjusted; model 2 adjusted for age and gender; model 3 adjusted for variable 2 in model 1, and chronic diseases, and all variables were adjusted in model 4, including age, gender, chronic diseases, education, marital status, smoking status, PIR, BMI, total daily energy, coffee intake, carbohydrate intake, serum Ca, race/ethnicity, alcohol use, physical activity, and Ca supplementation.

Statistically significant differences (*p* < 0.05), data with >20% effect on outcome depressive symptoms, variables that were not statistically significant but clinically significant for the occurrence of depressive symptoms. For the variable of Ca supplement, we used a dummy variable due to excessive missingness (>50%). Both variables were entered into the model simultaneously (Y = ax1 + b*dummy variable) (62).

To further explore the relationship between Ca intake and depressive symptoms, Ca intake (mg/day) was included in the model as a continuous variable. We used a restricted cubic spline curve for the analysis (63). Adjusting for all covariates, we chose the median dietary Ca as the reference value, which is not influenced by extreme values in the distribution series and is representative of the distribution series. Nodes were placed at the 5th, 35th, 65th, and 95th percentile. A two-sided *p* < 0.05 was considered statistically significant. Interactions between subgroups were examined by likelihood ratio tests.

## 3. Results

### 3.1. Baseline characteristics of the study populations

There were 14,971 people in this study, including 7,817 men and 7,154 women. We divided the population into four groups based on Ca intake. The lower Ca intake usually occurred in people who were older, female, non-smokers, non-alcohol drinkers, and had no diabetes. Those with higher Ca intakes were more likely to be married, male, better educated, and without serious CVD, as shown in Table 1.

**TABLE 1:** Baseline characteristic of participants according to calcium (Ca) quartiles groups.

Characteristics	Calcium (mg/day)					
	**Total**	**Q1^h^**	**Q2^i^**	**Q3^j^**	**Q4^k^**	***P*-value**
	**(*n* = 14,971)**	**(*n* = 3,576)**	**(*n* = 3,692)**	**(*n* = 3,846)**	**(*n* = 3,857)**	
Calcium (mg/day)		≤534	(535–817)	(818–1,190)	≥1,191	
Age, median (IQR^a^)	46.0 (32.0, 61.0)	49.0 (34.0, 63.0)	47.0 (33.0, 62.0)	46.0 (32.0, 60.0)	42.0 (30.0, 56.0)	<0.001*
Sex [*n* (%)]						<0.001*
Male	7,817 (52.2)	1,603 (44.8)	1,788 (48.4)	1,975 (51.4)	2,451 (63.5)	
Female	7,154 (47.8)	1,973 (55.2)	1,904 (51.6)	1,871 (48.6)	1,406 (36.5)	
Marital status [*n* (%)]						<0.001*
Married	7,742 (51.7)	1,703 (47.6)	1,936 (52.4)	2,041 (53.1)	2,062 (53.5)	
Widowed	847 (5.7)	276 (7.7)	208 (5.6)	217 (5.6)	146 (3.8)	
Divorced	1,624 (10.8)	452 (12.6)	418 (11.3)	400 (10.4)	354 (9.2)	
Separated	458 (3.1)	129 (3.6)	111 (3)	117 (3)	101 (2.6)	
Never married	2,997 (20.0)	716 (20)	710 (19.2)	761 (19.8)	810 (21)	
Living with partner	1,303 (8.7)	300 (8.4)	309 (8.4)	310 (8.1)	384 (10)	
Education [*n* (%)]						<0.001*
Below high school	2,958 (19.8)	882 (24.7)	715 (19.4)	690 (17.9)	671 (17.4)	
High school grad/GED^b^/equivalent	3,315 (22.1)	881 (24.6)	807 (21.9)	817 (21.2)	810 (21)	
Above high school	8,698 (58.1)	1,813 (50.7)	2,170 (58.8)	2,339 (60.8)	2,376 (61.6)	
Race/Ethnicity [*n* (%)]						<0.001*
Mexican American	2,108 (14.1)	423 (11.8)	517 (14)	555 (14.4)	613 (15.9)	
Other Hispanic	1,466 (9.8)	364 (10.2)	356 (9.6)	379 (9.9)	367 (9.5)	
Non-Hispanic white	6,942 (46.4)	1,362 (38.1)	1,678 (45.4)	1,841 (47.9)	2,061 (53.4)	
Non-Hispanic black	2,911 (19.4)	981 (27.4)	720 (19.5)	694 (18)	516 (13.4)	
Other races	1,544 (10.3)	446 (12.5)	421 (11.4)	377 (9.8)	3,080 (7.8)	
BMI^c^ [*n* (%)]						0.578
Under weight	221 (1.5)	58 (1.6)	62 (1.7)	52 (1.4)	49 (1.3)	
Normal weight	4,317 (28.8)	1,014 (28.4)	1,053 (28.5)	1,154 (30)	1,096 (28.4)	
Over weight	5,040 (33.7)	1,188 (33.2)	1,257 (34)	1,274 (33.1)	1,321 (34.2)	
Obese	5,393 (36.0)	1,316 (36.8)	1,320 (35.8)	1,366 (35.5)	1,391 (36.1)	
PIR^d^ [*n* (%)]						<0.001*
Poor	3,007 (20.1)	831 (23.2)	723 (19.6)	693 (18)	760 (19.7)	
Near poor	3,777 (25.2)	967 (27)	929 (25.2)	934 (24.3)	947 (24.6)	
Middle-income	3,958 (26.4)	949 (26.5)	978 (26.5)	1,055 (27.4)	976 (25.3)	
High-income	4,229 (28.2)	829 (23.2)	1,062 (28.8)	1,164 (30.3)	1,174 (30.4)	
Smoking status [*n* (%)]						<0.001*
Never	8,323 (55.6)	1,890 (52.9)	2,055 (55.7)	2,212 (57.5)	2,166 (56.2)	
Former	3,582 (23.9)	821 (23)	916 (24.8)	945 (24.6)	900 (23.3)	
Now	3,066 (20.5)	865 (24.2)	721 (19.5)	689 (17.9)	791 (20.5)	
Alcohol drinking [*n* (%)]						<0.001*
No	3,653 (24.4)	1,047 (29.3)	929 (25.2)	908 (23.6)	769 (19.9)	
Yes	11,318 (75.6)	2,529 (70.7)	2,763 (74.8)	2,938 (76.4)	3,088 (80.1)	
Carbohydrates (Mean ± SD^e^)	258.3 ± 122.5	186.2 ± 90.0	231.8 ± 94.8	267.3 ± 105.6	341.6 ± 136.0	<0.001*
Caffeine intake [Median (IQR)]	101.0 (15.0, 216.0)	91.0 (11.0, 198.0)	100.0 (20.0, 205.0)	101.0 (13.0, 216.0)	111.0 (18.0, 248.0)	<0.001*
Total energy intake (Mean ± SD)	2,148.8 ± 956.0	1,509.3 ± 645.2	1,923.9 ± 716.1	2,226.2 ± 790.1	2,879.9 ± 1,039.5	<0.001*
Calcium supplement [Median (IQR)]	315.0 (200.0, 600.0)	400.0 (210.0, 667.0)	383.0 (200.0, 627.5)	326.0 (200.0, 609.0)	238.0 (200.0, 600.0)	<0.001*
Serum vitamins D [*n* (%)]						<0.001*
<50 nmol/L	4,521 (30.2)	1,336 (37.4)	1,134 (30.7)	1,108 (28.8)	943 (24.4)	
50–74 nmol/L	5,612 (37.5)	1,269 (35.5)	1,343 (36.4)	1,430 (37.2)	1,570 (40.7)	
≥75 nmol/L	4,838 (32.3)	971 (27.2)	1,215 (32.9)	1,308 (34.0)	1,344 (34.8)	
Serum calcium (mmol/L) [Median (IQR)]	2.4 (2.3, 2.4)	2.4 (2.3, 2.4)	2.4 (2.3, 2.4)	2.4 (2.3, 2.4)	2.4 (2.3, 2.4)	<0.001*
Depression [*n* (%)]						<0.001*
No	13,827 (92.4)	3,220 (90)	3,419 (92.6)	3,587 (93.3)	3,601 (93.4)	
Yes	1,144 (7.6)	356 (10.0)	273 (7.4)	259 (6.7)	256 (6.6)	
Hypertension [*n* (%)]						<0.001*
No	10,156 (67.8)	2,270 (63.5)	2,472 (67.0)	2,647 (68.8)	2,767 (71.7)	
Yes	4,815 (32.2)	1,306 (36.5)	1,220 (33.0)	1,199 (31.2)	1,090 (28.3)	
Diabetes [*n* (%)]						<0.001*
No	1,530 (10.2)	449 (12.6)	402 (10.9)	367 (9.5)	312 (8.1)	
Yes	13,111 (87.6)	3,037 (84.9)	3,212 (87)	3,396 (88.3)	3,466 (89.9)	
Borderline	330 (2.2)	90 (2.5)	78 (2.1)	83 (2.2)	97 (2.0)	
CVD^f^ [*n* (%)]						<0.001*
No	14,028 (93.7)	3,283 (91.8)	3,449 (93.4)	3,615 (94)	3,681 (95.4)	
Yes	943 (6.3)	293 (8.2)	243 (6.6)	231 (6)	176 (4.6)	
Cancer/malignancy [*n* (%)]						0.188
No	13,644 (91.1)	3,263 (91.2)	3,342 (90.5)	3,495 (90.9)	3,544 (91.9)	
Yes	1,327 (8.9)	313 (8.8)	350 (9.5)	351 (9.1)	313 (8.0)	
Activity (MET^g^) [Median (IQR)]	315.0 (200.0, 600.0)	400.0 (400.0, 667.0)	383.0 (200.0, 627.5)	326.0 (200.0, 609.0)	238.0 (200.0, 600.0)	<0.001*

IQR^a^, interquartile range; GED^b^, general education development diploma; BMI^c^, body mass index; PIR^d^, the ratio of family income to poverty.

SD^e^, standard deviation; CVD^f^, severe cardiovascular diseases; MET^g^, metabolic equivalents of task.

Q1^h^, Ca intake is less than or equal to 534 mg/day.

Q2^i^, Ca intake ranges from greater than or equal to 535 and less than 817 mg/day.

Q3^j^, Ca intake ranges from greater than or equal to 818 and less than 1,190 mg/day.

Q4^k^, Ca intake ranges from greater than or equal to 1,191 mg/day.

*Significant difference among Ca quartiles groups as analyzed by single factor analysis or Kruskal–Wallis test (*p* < 0.05).

Table 2 showed the optimal Ca intake, according to the National Institutes of Health consensus conference. We compared the RDAs with the actual average Ca intake and found that the daily Ca intake of female, including pregnant and male aged of 18–24 and over 65, was lower than the recommended value, where the reference values for 18–24-year-olds and pregnant women were calculated by averaging the recommended dose range.

**TABLE 2:** Dietary calcium (Ca) among US adults (≥18 years) in National Health and Nutrition Examination Survey (NHANES) 2007–2016.

Age (year)	RDAs^c^ for calcium (mg/day)	Calcium intake (mg/day)	*P*
18–24^a^	1,350.00	1,207.6 (570.4)	<0.001
25–65^a^	1,000.00	1,078 (682.0)	<0.001
>65^a^	1,500.00	882.4 (497.9)	<0.001
18–24^b^	1,350.00	878.7 (518.6)	<0.001
25–50^b^	1,000.00	880.6 (494.0)	<0.001
>50^b^	1,500.00	813.4 (460.1)	<0.001
Pregnant	1,350.00	1,040.4 (570.4)	<0.001

^a^, Male; ^b^, female; RDA^c^, recommended dietary allowance.

The model-based ORs and 95% confidence intervals (CIs) are presented in Table 3. When not adjusted for covariates, each one-unit increase in Ca intake (1,000 mg/day) was associated with a 28% reduction in the risk of depressive infection (OR: 0.72; 95% CI: 0.63–0.81). We observed the same trend after adjusting for minimal model 2 sex, age, and model 3 chronic disease. when adjusting for all covariates in model 4, each one-unit increase in Ca intake was associated with an 18% reduction in the risk of depressive symptoms (OR: 0.82; 95% CI: 0.70–0.95).

**TABLE 3:** Association of calcium (Ca) and depression among participants in the NHANES^a^ 2007–2016 (*N* = 14,971).

Variable	Model I	*P*-value	Model II	*P*-value	Model III	*P*-value	Model IV	*P*-value
	**OR (95% CI)**		**OR (95% CI)**		**OR (95% CI)**		**OR (95% CI)**	
Calcium (1,000 mg/day)	0.72 (0.63∼0.81)	<0.001	0.77 (0.68∼0.88)	<0.001	0.8 (0.71∼0.91)	0.001	0.82 (0.70∼0.95)	0.011
**Subgroup (mg/day)**								
Q1^b^ (*n* = 3,576)	1 (Ref)		1 (Ref)		1 (Ref)		1 (Ref)	
Q2^c^ (*n* = 3,692)	0.72 (0.61∼0.85)	<0.001	0.73 (0.62∼0.87)	<0.001	0.76 (0.64∼0.9)	<0.001	0.83 (0.69∼0.99)	0.035
Q3^d^ (*n* = 3,846)	0.65 (0.55∼0.77)	<0.001	0.67 (0.57∼0.79)	<0.001	0.7 (0.59∼0.83)	<0.001	0.79 (0.65∼0.95)	0.012
Q4^e^ (*n* = 3,857)	0.64 (0.54∼0.76)	<0.001	0.71 (0.6∼0.84)	<0.001	0.75 (0.63∼0.89)	<0.001	0.80 (0.63∼0.98)	0.019
Trend.test		<0.001		<0.001		<0.001		0.014

Calculated using multivariate logistic regression analysis was performed.

Model I: no adjusted. Model II: adjusted for age + sex.

Model III: model II + hypertension + diabetes + cardiovascular disease (CVD) + cancer/malignancy.

Model IV: model III + race/ethnicity + education + marital status + poverty to income ratio (PIR) + body mass index (BMI) + smoking status + drinking status + activity + carbohydrates intake + caffeine intake + total energy intake + serum Ca + serum vitamins D + Ca supplement.

NHANES^a^, National Health and Nutrition Examination Survey.

Q1^b^, Ca intake is less than or equal to 534 mg/day.

Q2^c^, Ca intake ranges from greater than or equal to 535 and less than 817 mg/day.

Q3^d^, Ca intake ranges from greater than or equal to 818 and less than 1,190 mg/day.

Q4^e^, Ca intake ranges from greater than or equal to 1,191 mg/day.

**FIGURE 2:**
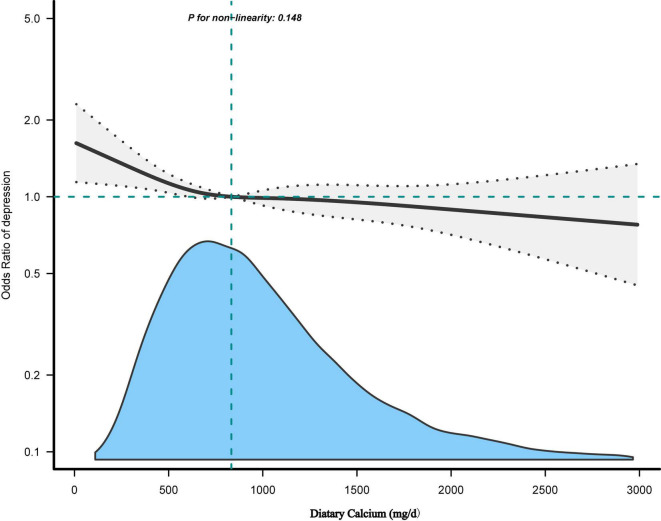
The dose-response relationship between dietary calcium (Ca) intake and the risk of depressive symptoms. Adjusted for: Age + sex + hypertension + diabetes + CVD + cancer/malignancy + race/ethnicity + education + marital status + poverty to income ratio (PIR) + body mass index (BMI) + smoking status + drinking status + activity + carbohydrates intake + caffeine intake + total energy intake + serum Ca + serum vitamins D + Ca supplement.

Table 3 showed the effect values were less than 1 between Ca quartiles and depression in model 1 (Q2; OR, 0.72; 95% CI, 0.61–0.85; Q3; OR, 0.65; 95% CI, 0.55–0.77, and Q4; OR, 0.64; 95% CI, 0.54–0.76) compared with Q1 (<534 mg/day). After adjusting all variables, the same trend was observed in adjusted model 2, model 3, and model 4. The results were similar in all participants who did not exclude very high dietary Ca (Supplementary Table 1) and in the weighted analysis (Supplementary Table 2). In restricted cubic curve spline analysis, we observed a linear relationship between the continuous variable Ca intake and depressive symptoms (non-linear *p* = 0.148) in Figure 2.

### 3.2. Sensitivity analysis

**FIGURE 3:**
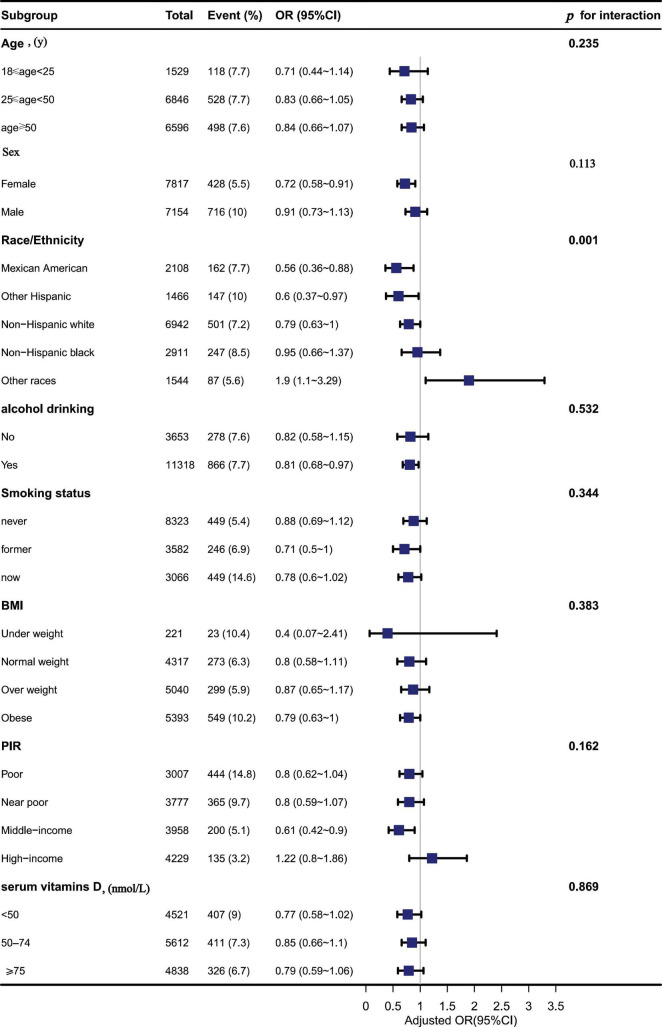
The association between dietary calcium (Ca) intake and depressive symptoms in different subgroups.

In these subgroups, we adjusted the full model and found an interaction between Ca and depressive symptoms in races (*p* = 0.001), with Ca intake likely to reduce depressive symptoms in Mexican Americans, while Ca intake may increase depressive symptoms in other races (OR: 1.9; 95% CI, 1.10–3.29) in Table 4. No interactions, however, were found in other subgroups (*p* > 0.05) in Figure 3.

**TABLE 4:** Association between depression and calcium (Ca) intake in subgroups.

Subgroups	Case/Participants (%)	Crude OR^a^ (95% CI^b^)	*p*	Adjust OR (95% CI)	*p*	*P* for interaction
Age (years)						0.235
≥18 < 25	118/1,529 (7.7)	0.67 (0.47∼0.96)	0.029	0.71 (0.44∼1.14)	0.153	
≥25 < 50	528/6,846 (7.7)	0.64 (0.53∼0.76)	<0.001	0.83 (0.66∼1.05)	0.125	
≥50	498/6,596 (7.6)	0.84 (0.69∼1.03)	0.09	0.84 (0.66∼1.07)	0.164	
Sex						0.113
Male	428/7,817 (5.5)	0.72 (0.59∼0.86)	<0.001	0.72 (0.58∼0.91)	0.006	
Female	716/7,154 (10.0)	0.85 (0.72∼1.01)	0.068	0.91 (0.73∼1.13)	0.400	
Race/Ethnicity						0.001
Mexican American	162/2,108 (7.7)	0.54 (0.39∼0.76)	<0.001	0.56 (0.36∼0.88)	0.011	
Other Hispanic	147/1,466 (10.0)	0.49 (0.33∼0.71)	<0.001	0.6 (0.37∼0.97)	0.037	
Non-Hispanic white	501/6,942 (7.2)	0.69 (0.57∼0.83)	<0.001	0.79 (0.63∼1.00)	0.052	
Non-Hispanic black	247/2,911 (8.5)	0.93 (0.7∼1.23)	0.607	0.95 (0.66∼1.37)	0.784	
Other races	87/1,544 (5.6)	1.56 (1.04∼2.34)	0.031	1.9 (1.10∼3.29)	0.022	
Alcohol drinking						0.532
No	278/3,653 (7.6)	0.76 (0.59∼0.99)	0.045	0.82 (0.58∼1.15)	0.254	
Yes	866/11,318 (7.7)	0.70 (0.61∼0.81)	<0.001	0.81 (0.68∼0.97)	0.021	
Smoking status						0.344
Never	449/8,323 (5.4)	0.8 (0.66∼0.97)	0.026	0.88 (0.69∼1.12)	0.311	
Former	246/3,582 (6.9)	0.72 (0.55∼0.94)	0.017	0.71 (0.50∼1.00)	0.050	
Now	449/3,066 (14.6)	0.70 (0.57∼0.85)	<0.001	0.78 (0.60∼1.02)	0.065	
BMI^c^						0.383
Under weight	23/221 (10.4)	0.94 (0.4∼2.21)	0.893	0.4 (0.07∼2.41)	0.320	
Normal weight	273/4,317 (6.3)	0.65 (0.5∼0.85)	0.001	0.80 (0.58∼1.11)	0.190	
Over weight	299/5,040 (5.9)	0.80 (0.63∼1.01)	0.062	0.87 (0.65∼1.17)	0.347	
Obese	549/5,393 (10.2)	0.71 (0.59∼0.85)	<0.001	0.79 (0.63∼1.00)	0.048	
PIR^d^						0.162
Poor	444/3,007 (14.8)	0.74 (0.61∼0.9)	0.003	0.80 (0.62∼1.04)	0.094	
Near poor	365/3,777 (9.7)	0.70 (0.56∼0.87)	0.001	0.80 (0.59∼1.07)	0.126	
Middle-income	200/3,958 (5.1)	0.72 (0.53∼0.98)	0.035	0.61 (0.42∼0.90)	0.011	
High-income	135/4,229 (3.2)	1.07 (0.77∼1.48)	0.697	1.22 (0.8∼1.86)	0.352	
Serum vitamins D (nmol/L)						0.869
<50	407/4,521 (9.0)	0.75 (0.6∼0.93)	0.008	0.77 (0.58∼1.02)	0.064	
50–74	411/5,612 (7.3)	0.74 (0.61∼0.90)	0.003	0.85 (0.66∼1.10)	0.220	
≥75	326/4,838 (6.7)	0.72 (0.57∼0.91)	0.006	0.79 (0.59∼1.06)	0.120	

OR^a^, odds ratio; 95% CIs^b^, 95% confidence intervals; BMI^c^, body mass index; PIR^d^, the ratio of family income to poverty. All subgroups had adjusted for Age + sex + hypertension + diabetes + cardiovascular disease (CVD) + cancer/malignancy + race/ethnicity + education + marital status + poverty to income ratio (PIR) + BMI + smoking status + drinking status + activity + carbohydrates intake + caffeine intake + total energy intake + serum Ca + serum vitamins D + Ca supplement (Model IV).

## 4. Discussion

The results suggest an inverse association between dietary Ca intake and the risk of depressive symptoms in the US population >18 years of age. This association was maintained even after adjusting for a large number of potential confounders such as gender, age, race, education, smoking, physical activity, alcohol consumption, marital status, BMI, history of severe CVD, history of hypertension, history of diabetes, cancer or malignancy, Ca supplements, serum Ca, serum vitamin D, caffeine, carbohydrate, and total energy intake. We also found that U.S. adults are eating less Ca than RDAs. Therefore, American adults need to increase their awareness of Ca intake, which may have some benefits in preventing depressive symptoms.

In the cross-sectional study, there is no significant association between dietary Ca intake and risk of depressive symptoms was detected (64). First of all, Joseph R’s study subjects were 279 women at home, and the small sample size compared with ours may lead to the different result from our study. Furthermore, self-reported Older American Nutrition Act Program (OAANP) home delivery services were not able to keep up with the dietary needs of elderly women at home, and 32% of the sample subjects were at risk of insufficient food, which may have influenced their findings. Therefore, differences in study settings and samples may explain the inconsistent results to some extent. However, our results are consistent with those of the previous several cross-sectional studies. In these studies, Ca intake was associated with a lower incidence of depression (65, 66).

The following study can also indirectly support our results. Bae et al. (67) found that pregnant women in the low depression level group had a significantly higher intake of total Ca and plant Ca than those in the high depression level group. A study from Zhejiang’s major public health surveillance program of 8,527 elderly people over 60 years old showed that oral vitamin D supplementation and Ca or Ca alone could relieve depression (68).

The inverse association between Ca intake and depression can be explained by several potentially related mechanisms. First, Ca is involved in regulating the hypothalamic-pituitary-adrenal (HPA) system, which is considered the body’s primary stress response system (69). Corticotropin-releasing hormone (CRH) stimulates the hypothalamus to secrete adrenocorticotropic hormone (ACTH) regulating the release of adrenocorticosteroid, the HPA system is regulated by CRH (70, 71). Therefore, If there is a dysregulation between the CRH and HPA systems, this would further affect stress hormones, such as cortisol, which will affect depressive symptoms (72).

Second, extracellular Ca influx is an important component of many neuronal processes (73). The change of extracellular Ca^2+^ concentration may participate in emotion regulation, which may be the direct effect of Ca on stabilizing the plasma membrane, and methyl-D-aspartate may also affect neural plasticity (74). Ca/calmodulin-dependent protein kinase II has been reported to mediate the process of metabotropic glutamate receptor production activity in group I of the rat hippocampus, leading to chronic depression (75, 76). Ca activates tryptophan hydroxylase in biosynthetic pathways leading to serotonin synthesis, while cytosolic Ca concentration plays a key role in stimulus-response coupling in various tissues, and disturbances in regulation may have a broad impact on cellular function, which in turn may alter mood (77, 78).

Our study has several strengths. First, to our knowledge, this is one of the few studies that have examined dietary Ca and depressive symptoms alone. In addition, we evaluated the dose relationship between Ca and depressive symptoms in addition to the corresponding sensitivity analysis. However, there are some limitations. Firstly, our study is a cross-sectional study in an observational study and therefore cannot account for causality. Secondly, using the 24-h dietary recall interview for dietary assessment can overestimate or underestimate actual intake. But the way to collect dietary data had been shown to be effective (79). Thirdly, the depressive symptoms in study were based on self-reports, therefore, it may be subject to misunderstanding of the question. Finally, although we have controlled for potential confounders as we have learned about, there may be additional covariates that were not included. However, there is no doubt that our findings contribute to the research on the link between Ca intake and depressive symptoms.

## 5. Conclusion

In conclusion, our results suggest an association between dietary Ca and the prevalence of depressive symptoms in United States adults. And Ca intake was negatively associated with the risk of depressive symptoms. As Ca intake increased, the prevalence of depressive symptoms decreased. More prospective studies are needed to examine the relationship between Ca and depressive symptoms.

## Data availability statement

The datasets presented in this study can be found in online repositories. The names of the repository/repositories and accession number(s) can be found in the article/Supplementary material.

## Ethics statement

The survey protocol for the NHANES was approved by the CDC’s National Center for Health Statistics Institutional Research Ethics Review Board. All participants provided written informed consent, and the study was approved by the NCHS Research Ethics Review Board (https://wwwn.cdc.gov/nchs/nhanes/default.aspx).

## Author contributions

XS designed the research, conducted the statistical analyses, and drafted the manuscript. XS, XG, Y-YL, and LY completed the final draft. MZ and LJ reviewed the manuscript and supervised. All authors have made substantial contributions to the work, read the manuscript, agreed on the submission of this work to the journal, accept responsibility for the manuscript’s contents, and finalized the manuscript.
