# Supplementary material for: Association between dietary calcium and depression among American adults: National health and nutrition examination survey
Source: Front Nutr. 2023 Feb 9;10:1042522. doi: 10.3389/fnut.2023.1042522 (PMC9948022; doi:10.3389/fnut.2023.1042522)
Supplement: Supplementary file 1 [file Table_1.DOCX]

| **Supplementary Table S1: Association of calcium and depressive symptoms among participants who did not exclude very high dietary calcium intake in the NHANES ^a^ 2007-2016. (n=15162)** | | | | | | | | | | |
| --- | --- | --- | --- | --- | --- | --- | --- | --- | --- | --- |
| **Variable** | **Model Ⅰ** | **P value** | **Model Ⅱ** | **P value** | **Model Ⅲ** | **P value** | **Model Ⅳ** | **P value** | **Model Ⅴ** | **P value** |
|  | **OR (95%CI)** |  | **OR (95%CI)** |  | **OR (95%CI)** |  | **OR (95%CI)** |  | **OR (95%CI)** |  |
| **calcium（g/d）** | 0.79 (0.71~0.89) | <0.001 | 0.85 (0.76~0.95) | 0.005 | 0.88 (0.79~0.98) | 0.025 | 0.91 (0.79~1.05) | 0.185 | 0.89 (0.78~1.03) | 0.112 |
| **subgroup（mg/d）** | |  |  |  |  |  |  |  |  |  |
| **Q1^b^ (n = 3576)** | 1(Ref) |  | 1(Ref) |  | 1(Ref) |  | 1(Ref) |  | 1(Ref) |  |
| **Q2^c^ (n = 3692)** | 0.72 (0.61~0.85) | <0.001 | 0.76 (0.67~0.86) | <0.001 | 0.79 (0.7~0.89) | <0.001 | 0.84 (0.71~1.01) | 0.06 | 0.83 (0.7~0.99) | 0.04 |
| **Q3^d^ (n = 3846)** | 0.65 (0.55~0.77) | <0.001 | 0.74 (0.65~0.84) | <0.001 | 0.77 (0.68~0.88) | <0.001 | 0.81 (0.67~0.97) | 0.024 | 0.79 (0.66~0.95) | 0.014 |
| **Q4^e^ (n = 4012)** | 0.66 (0.56~0.77) | <0.001 | 0.75 (0.66~0.85) | <0.001 | 0.79 (0.69~0.9) | <0.001 | 0.83 (0.67~1.01) | 0.067 | 0.8 (0.65~0.98) | 0.034 |
| **Trend .test** |  | <0.001 |  | <0.001 |  | <0.001 |  | 0.048 |  | 0.024 |

Calculated using multivariate logistic regression analysis was performed;

Model Ⅰ: no adjusted;

Model Ⅱ: adjusted for Age +sex;

Model Ⅲ: Mode Ⅱ +hypertension + diabetes + CVD + cancer /malignancy;

Model Ⅳ: Mode Ⅲ + education +marital status +PIR +smoking status + BMI +Carbohydrates intake+ caffeine intake +Total energy intake +serum calcium +serum vitamins D;

Model Ⅴ: Model Ⅳ +race/ ethnicity +drinking status +calcium supplement +activity;

Abbreviations: NHANES ^a^, National Health, and Nutrition Examination Survey.

Q1^b^, calcium intake is less than or equal to 534 mg/d;

Q2^c^, calcium intake ranges from greater than or equal to 535 and less than 817 mg/d;

Q3^d^, calcium intake ranges from greater than or equal to 818 and less than 1190 mg/d;

Q4e, calcium intake ranges from greater than or equal to 1191mg/d.

| **Supplementary Table S2:** **Weighted logistic regression between calcium and depressive symptoms among participants in the NHANES ^a^ 2007-2016. (N=14971)** | | | | | | | | |
| --- | --- | --- | --- | --- | --- | --- | --- | --- |
| **Variable** | **Model Ⅰ** | ***P* value** | **Model Ⅱ** | ***P* value** | **Model Ⅲ** | ***P* value** | **Model Ⅳ** | ***P* value** |
|  | **OR (95%CI)** |  | **OR (95%CI)** |  | **OR (95%CI)** |  | **OR (95%CI)** |  |
| **calcium（1000mg/d）** | 0.68 (0.580~0.794) | <0.001 | 0.73 (0.616~0.861) | <0.001 | 0.75(0.637~0.892) | 0.001 | 0.86 (0.720~0.957) | 0.006 |
| **subgroup（mg/d）** | |  |  |  |  |  |  |  |
| **Q1^b^(n=3576)** | 1(Ref) |  | 1(Ref) |  | 1(Ref) |  | 1(Ref) |  |
| **Q2^c^ (n=3692)** | 0.74 (0.657~0.829) | <0.001 | 0.75(0.667~0.842) | <0.001 | 0.77(0.678~0.864) | <0.001 | 0.83 (0.724~0.945) | 0.005 |
| **Q3^d^ (n=3846)** | 0.67 (0.593~0.753) | <0.001 | 0.69 (0.617~0.783) | <0.001 | 0.71 (0.626~0.804) | <0.001 | 0.79 (0.691~0.916) | 0.001 |
| **Q4^e^ (n=3857)** | 0.66 (0.584~0.741) | <0.001 | 0.73(0.645~0.822) | <0.001 | 0.77(0.682~0.879) | <0.001 | 0.84(0.717~0.987) | 0.034 |
| **Trend .test** |  | <0.001 |  | <0.001 |  | <0.001 |  | 0.017 |

Calculated using multivariate logistic regression analysis was performed;

Model Ⅰ: no adjusted;

Model Ⅱ: adjusted for Age +sex;

Model Ⅲ: Mode Ⅱ +hypertension + diabetes + CVD + cancer /malignancy;

Model Ⅳ: Mode Ⅲ + education +marital status +PIR +smoking status + BMI +Carbohydrates intake+ caffeine intake +Total energy intake +serum calcium +serum vitamins D;

Model Ⅴ: Model Ⅳ +race/ ethnicity +drinking status +calcium supplement +activity;

Abbreviations: NHANES ^a^, National Health, and Nutrition Examination Survey.

Q1^b^, calcium intake is less than or equal to 534 mg/d;

Q2^c^, calcium intake ranges from greater than or equal to 535 and less than 817 mg/d;

Q3^d^, calcium intake ranges from greater than or equal to 818 and less than 1190 mg/d;

Q4e, calcium intake ranges from greater than or equal to 1191mg/d.
